# Finite Element Analysis of the Structure and Working Principle of Solid-State Shear Milling (S3M) Equipment

**DOI:** 10.3390/ma17174210

**Published:** 2024-08-26

**Authors:** Lingfei Wei, Chao Wang, Ruoxuan Duan, Zehang Zhou, Canhui Lu

**Affiliations:** 1 State Key Laboratory of Polymer Materials Engineering, Polymer Research Institute, Sichuan University, Chengdu 610065, China; wlflingfei@163.com (L.W.); 2021223090003@stu.scu.edu.cn (R.D.); 2 National Engineering Research Center for Synthesis of Novel Rubber and Plastic Materials, Beijing Research Institute of Chemical Industry, SINOPEC, Beijing 100013, China; wangchao.bjhy@sinopec.com

**Keywords:** solid-state shear milling equipment, finite element method, pan-milling, material processing, mechanochemical reactions

## Abstract

Solid-state shear milling (S3M) equipment is an evolution from traditional stone mills, enabling the processing of polymer materials and fillers through crushing, mixing, and mechanochemical reactions at ambient temperature. Due to the complex structure of the mill-pan, empirical data alone are insufficient to give a comprehensive understanding of the physicochemical interactions during the milling process. To provide an in-depth insight of the working effect and mechanism of S3M equipment, finite element method (FEM) analysis is employed to simulate the milling dynamics, which substantiates the correlation between numerical outcomes and experimental observations. A model simplification strategy is proposed to optimize calculation time without compromising accuracy. The findings in this work demonstrate the S-S bond breakage mechanism behind stress-induced devulcanization and suggest the structural optimizations for enhancing the devulcanization and pulverization efficiency of S3M equipment, thereby providing a theoretical foundation for its application in material processing.

## 1. Introduction

**Figure 1:**
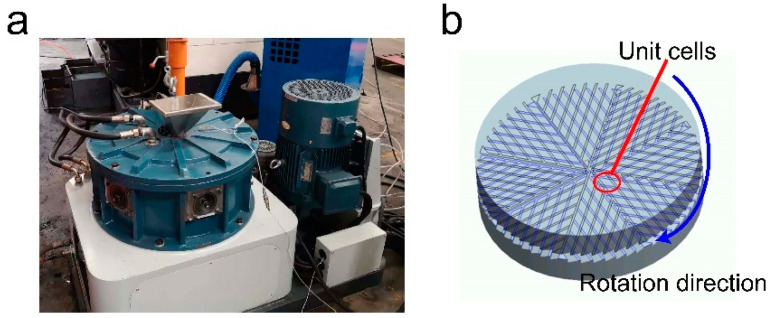
Schematic diagram of S3M equipment and the structure of the inlaid pans. (**a**) S3M equipment. (**b**) Schematic diagram of the unit cells formed by ridges and bevels on the moving pan and stationary pan.

The origin of milling equipment can be traced back to the stone mill [1], a pivotal invention in early agricultural societies, which has been used to mill rice, wheat, corn, and other crops for thousands of years. The structural design of its grinding teeth and grooves has been continuously improved [2,3] to adapt to the growing production requirements [4], ranging from crops to various materials [5,6]. The invention of solid-state shear milling (S3M), with the advantages of high efficiency and high throughput, represents a significant advancement in this evolution, employing principles of polymer mechanochemistry to facilitate the crushing, mixing, and mechanochemical reactions of polymer materials and fillers [7] (Figure 1a,b). The development of this innovative technology requires a comprehensive analysis of its operational mechanisms and the optimization of its design for enhanced efficiency.

The mill-typed inlaid pans of S3M equipment, which are made of wear-resistant alloy and drawn from traditional Chinese stone mills, are divided into eight equal sectors by several diameter lines. Each sector is divided equally by several parallel sloping surfaces. The moving pan and stationary pan have similar structures and are placed together, face-to-face, and the ridges and bevels on both pans form many unit cells (Figure 1b). They are changed in shape and size as they move outward with the rotation of the moving pan.

First introduced by Ostwald [8], mechanochemistry demonstrates a paradigm shift in chemical synthesis to induce chemical reactions by stress, which is different from the traditional chemical reactions initiated by catalysis, heat, light, and electricity [9]. Polymer mechanochemistry unveils the potential of stress-induced reactions in polymers with the breakage of chemical bonds and generation of macromolecular radicals, which minimizes the solvent use and enables the reactions unfeasible in solution. Specifically, it provides a new pathway for the preparation and modification of polymer materials, which has important academic significance and practical application value [10]. Previous research work has shown that S3M equipment can provide powerful extrusion, tension, and three-dimensional shear force fields [4,11] with a variety of effects, such as comminution, dispersion, mixing, and force activation [12,13], which is feasible to pulverize viscoelastic and thermosensitive general-purpose polymers, engineering plastics, special engineering plastics, and biopolymer materials at room temperature into micron-nanometer-sized ultrafine powders [14,15]. Under the strong stress of S3M equipment, solid-phase uniform mixing and compatible dispersion are achieved with the co-milling of thermodynamically incompatible and viscosity-mismatched polymers [16]. S3M is also a valid strategy for the in situ surface modification of polymer materials [17,18]. Despite these achievements, a detailed and in-depth understanding of the force analysis of the grounded materials, the milling process, and the underlying material crushing principles remains elusive. Although Xu et al. [7] developed a mathematical model of the structure of the mill-pan and the velocity and trajectory of the particle motion, the difficulty remains due to the complexity of the mill-pan’s structure and the dynamic nature of the milling process, which poses a significant challenge to the optimization of S3M equipment for broader material processing applications.

This work aims to give an understanding of the milling process of the S3M strategy using the finite element method (FEM). A model simplification strategy was proposed to greatly reduce the calculation time while ensuring the accuracy of the calculation. Through finite element analysis, a numerical model of S3M was established to elucidate the structure–function relationship of the mill-pan in material processing. In addition, a structural analysis of the inlaid pans was conducted to give in-depth insights of the key component of the equipment innovation. This work provides a theoretical basis for optimizing the milling process and the structural parameters of the mill-pan, which can help to improve the processing efficiency and broaden the application of S3M equipment.

## 2. Experimental

### 2.1. Materials

Polystyrene pellets, iron pellets, and 316L stainless steel pellets were purchased from Chengdu Kelong Company (Chengdu, China). The three materials had particle sizes ranging from 100 to 600 μm.

### 2.2. S3M Processing of the Samples

The primary experimental apparatus utilized is the solid-state shear milling (S3M) equipment, meticulously devised and fabricated by the State Key Laboratory of Polymer Materials Engineering (Chengdu, China). This advanced equipment comprises a pair of precision-engineered milling surfaces and a sophisticated transmission system with rotational speed that is finely tuned through gear reduction mechanisms.

The key component of this innovative equipment is the inlaid pans, crafted from wear-resistant metal and specially treated on their surfaces. The stationary pan is fixed to the equipment case, while the moving pan is mounted on an axis, driven by an electric motor with a power range of 2–12 kW, depending on the equipment mode. The speed of the moving pan can be adjusted from 10 to 100 r min^−1^. The pressure exerted on the pans is adjustable via a screw system, ranging from 800 to 10^4^ N, determined using a stress–strain testing system. The equipment operates within a sealed system, allowing for the introduction of a cooling or heating medium and nitrogen gas through various inlets. Materials are fed into the equipment through an inlet, ground by the mill pans, and proceed through a soft tube to exit through an outlet, completing one milling cycle. The operation is continuous, enabling materials to be milled repeatedly based on final product requirements. The pan mill type equipment is designed with various configurations, including a horizontal axis, a vertical axis, series mounting pans, parallel mounting pans, and series–parallel mounting pans [7].

### 2.3. Characterization

Field-emission scanning electron microscopy (FESEM, JEOL JSM-7500F, Tokyo, Japan) was used to evaluate the surface morphology. Particle size was determined using a laser particle sizer (Malvern Panalytical Ltd., Malvern, UK) for precise characterization.

### 2.4. Modelling

The model in the simulation system comprises two parts: the material, typically in granular or powdered form, and the mill-pan. For the modelling of granular or powder, the most straightforward approach is to represent each particle as a spherical element. However, this modelling strategy poses significant computational challenges. Firstly, a small quantity of powder may contain thousands of particles, which might result in an exponential increase in contact relationships with its intricate web of contact, collision, and friction interactions among components. This complexity far exceeds the processing capabilities of current computing resources, rendering the simulation of a finite element model with extensive components and interactions impractical. Secondly, due to the small size of the components (generally several hundred microns) [14,15], the time step would have to be set small enough to satisfy the stability condition of the explicit integral equation. However, it would possibly require hundreds of millions of time steps [19]. This requirement compounds the computational difficulty, especially when considering complex phenomena like particle fragmentation.

In addition to the aforementioned computational difficulties, the surface energy of the particles [12] and the friction coefficient may also introduce further variables. The superposition of these issues makes it necessary to simplify the model. The primary objective of this work is to elucidate the stress field that S3M equipment imparts on the material and to understand its working process and mechanism. Therefore, the complex interactions between particles are deemed secondary, allowing for the powder to be considered as a collective entity for finite element analysis.

**Figure 2:**
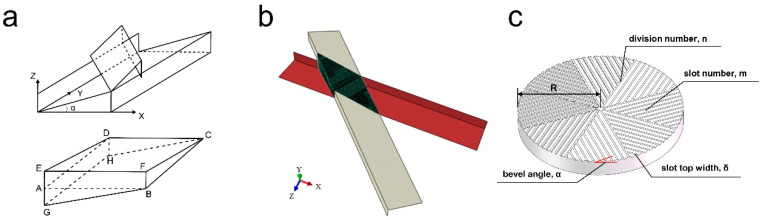
Schematic diagram of the mill-pan structure. (**a**) Schematic diagram of two bevels of mill-pan (upper) and unit cells (lower). (**b**) Simplified models for finite element analysis. (**c**) Structure of the inlaid pan.

Based on the 8-fold symmetry of the material model and the similarity of the individual cell units, only one unit was selected for analysis. The material model between the two mill-pans is obtained by Boolean operation of the 3D graph. In the finite element analysis, the strain of the mill-pan during pan-milling is negligible, and the mill-pan is not a force object in the finite element analysis but only plays a role in applying external forces. Therefore, the mill-pan can be simplified to two sets of rigid surfaces in contact with the material. This is consistent with the structural model of S3M equipment established by Xu et al. [7] (Figure 2a).

The feeding port of S3M equipment is located at the center of the mill-pan. The unit cells around the feeding port are easy to fill and accumulate particles due to the low linear velocity and fast feeding rate. Therefore, we consider the particles in the unit cell near the inlet as a whole. The mill-pan is crafted from a wear-resistant alloy with negligible strain during milling, which is considered in terms of two rigid surfaces interacting with the material, thus simplifying the model (Figure 2b). The main structural parameters of the mill-pan are the radius R, the division number n, the slot number m on each sector, the bevel angle α, and the slot top width δ (Figure 2c). The structural parameters of the mill-pan are shown in Table 1. The 3D model of the mill-pan with varying configurations can be constructed by adjusting these important parameters. The material model between the two mill-pans is obtained by Boolean operation of the 3D graph. Based on the 8-fold symmetry principle of the mill, only one-eighth of the material is modeled, reducing the computational requirements. For more information on the simplified model building process, see Supplementary Materials.

In this paper, AISI 1020 stainless steel is used as the abrasive material, and the mechanical parameters of the material are obtained from the material database of the software (Abaqus 6.14) [20,21]. Boundary condition settings, 3D model design, calculation methods, and mesh division in the finite element simulation are reflected in the Supplementary Materials [22,23].

## 3. Results and Discussion

**Figure 3:**
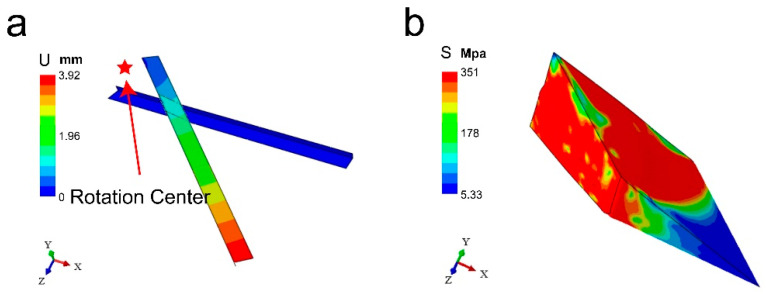
Finite element analysis calculation results. (**a**) Displacement diagram and (**b**) von Mises stress field.

Figure 3 illustrates the finite element simulation results of the effects of pan-milling on the materials. The displacement diagram shows that the stationary pan is completely fixed with zero displacement, while the groove of the moving pan makes a circular motion around the center of the mill-pan. This motion generates a gradient in linear velocity—and consequently, displacement—with values increasing with distance from the center, leading to a stepwise distribution pattern in displacement magnitudes. Figure 3b shows the von Mises stress distribution diagram within the material under the external force, with the red area indicating that the Mises stress on the material has reached the yield limit. According to the Mises yield criterion, a material unit starts to undergo plastic deformation when its equivalent Mises stress reaches the yield limit. If it is assumed that damage occurs when the material reaches the yield point, the Mises stress distribution can be used as an indicator for potential yield-induced damage (See the Supplementary Materials for details).

The simulation outcomes show that a large portion of the material is subjected to a Mises stress of 351.571 MPa, which reaches the predetermined yield stress for the material. According to the Mises yield criterion and the assumption that material damage initiates at the yield threshold, it can be inferred that the three-dimensional scissor structure of the mill-pan can effectively crush the material during grinding.

In order to verify the reliability of the numerical simulation, we used S3M equipment for the grinding of polystyrene, iron, and 316L [24] stainless steel particles, respectively, and the basic parameters of the three materials are shown in Table 2. This empirical approach not only substantiates the reliability of the simulation results but also underscores the effectiveness of the S3M equipment in processing different materials.

**Figure 4:**
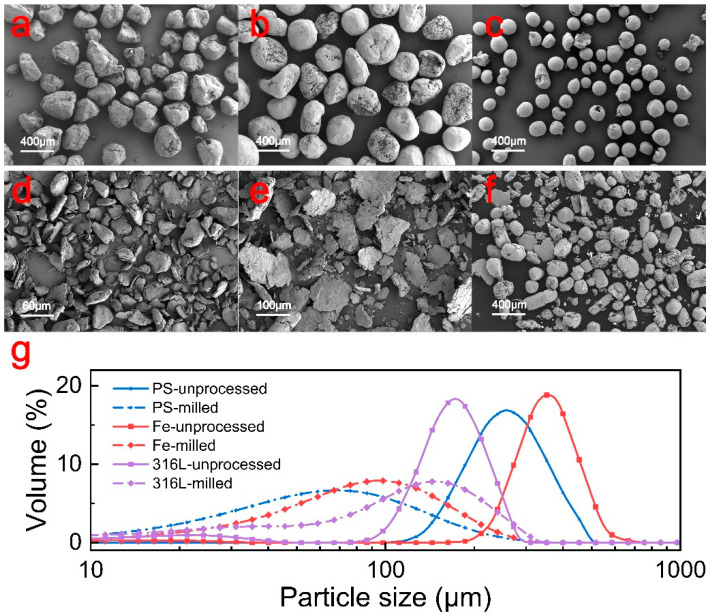
Characterization of particle morphology and particle size. (**a**–**c**) Scanning electron micrograph of unprocessed PS, Fe, and 316L stainless steel. (**d**–**f**) Scanning electron micrograph of milled PS, Fe, and 316L stainless steel. (**g**) Particle size distribution of three different materials before and after pan-milling.

Figure 4 shows the scanning electron microscope (SEM) photographs and particle size distribution of the polystyrene, iron, and 316L stainless steel particles before and after pan-milling. The shape of these particles before pan-milling is close to spherical. The particle sizes of the polystyrene, iron, and 316L stainless steel particles are approximately 257 μm, 350 μm, and 171 μm, respectively, and the particle size distribution is narrow. The pan-milling process exerted compression and shearing forces on the particles, leading to the fragmentation of larger particles into smaller ones, thereby reducing the average particle sizes to 68 μm, 97 μm, and 147 μm, respectively, and broadening their size distributions. By examining the particle size distribution graphs and SEM images, it becomes evident that the sizes of the polystyrene and iron particles decrease significantly. This further suggests their transformation into finer fragments during pan-milling. Notably, the ductile nature of iron facilitates its transformation into both smaller fragments and lamellar shapes. For comparison, the particle size change in the 316L stainless steel particles before and after pan-milling is small. Most steel particles are only deformed after pan-milling instead of breaking into small particles. This could be attributed to the fact that the S3M equipment is primarily designed to process polymer material, which is not suitable for processing materials with similar high hardness due to the milling apparatus’s material constraints.

Both experimental results and finite element analysis demonstrate the strong stress field exerted by the unique structure of S3M equipment, which can be used to pulverize polymer materials and produce ultrafine or even nano-sized powders from large polymer and metal particles. In addition, S3M equipment also has obvious advantages in terms of processing efficiency over traditional mechanochemical methods, such as high-energy ball milling, whose crushing efficiency depends on the collision probability between the material and the milling media (balls), the impact force, and the oscillation frequency and amplitude of the equipment. Pentimalli et al. [29] report an output of approximately 250 g of ABS powder per hour with a particle size of approximately 1 mm using high-energy ball milling. For comparison, S3M equipment with a unique structure can exert intense shear stress and pressure directly on the material. The direct contact between the crushing medium (mill-pan) and the crushed material results in high crushing efficiency. The mass of the three granular materials in this experiment is 1 kg, while the milling time is only approximately 5 min, demonstrating that S3M equipment has superior efficiency over the high-energy ball mill.

**Figure 5:**
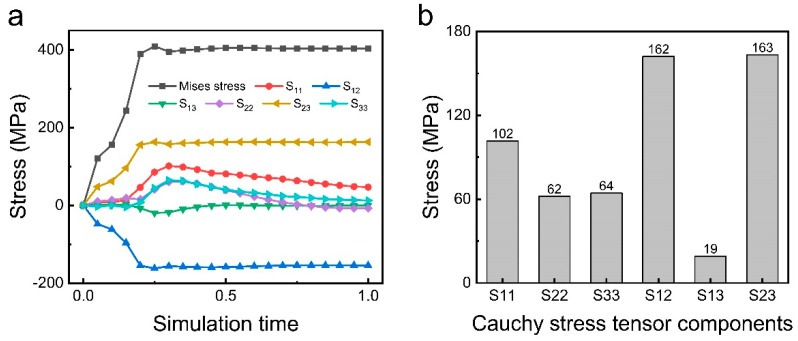
Stress analysis of a node in finite element analysis. (**a**) Variation in Mises stress and its stress components with time. (**b**) The magnitude of the Cauchy stress tensor components.

The crushing of materials during pan-milling results from a synergistic application of multiple stresses, including strong compression, three-dimensional shear, friction, and circumferential stresses. The determination of the predominant stress form responsible for the crushing of material requires further analysis of the directional stress component’s role. The Mises stress is decomposed into six Cauchy stress tensor components according to the spatial coordinate system (Figure 5a,b), where S_11_, S_22_, and S_33_ represent the positive stresses along the x, y, and z axes, respectively, and S_12_, S_13_, and S_23_ represent the shear stresses in the xy, xz, and yz planes, respectively. Based on Figure 5, it is evident that the absolute values of S_12_ and S_23_, denoting the shear stresses in the xy and yz planes, respectively, are notably higher. This suggests that the mill-pan structure can exert significant shear stress on the material, thereby serving as the primary mechanism for material crushing. This conclusion highlights shear stress as the principal mechanism underlying material fragmentation during pan-milling.

**Figure 6:**
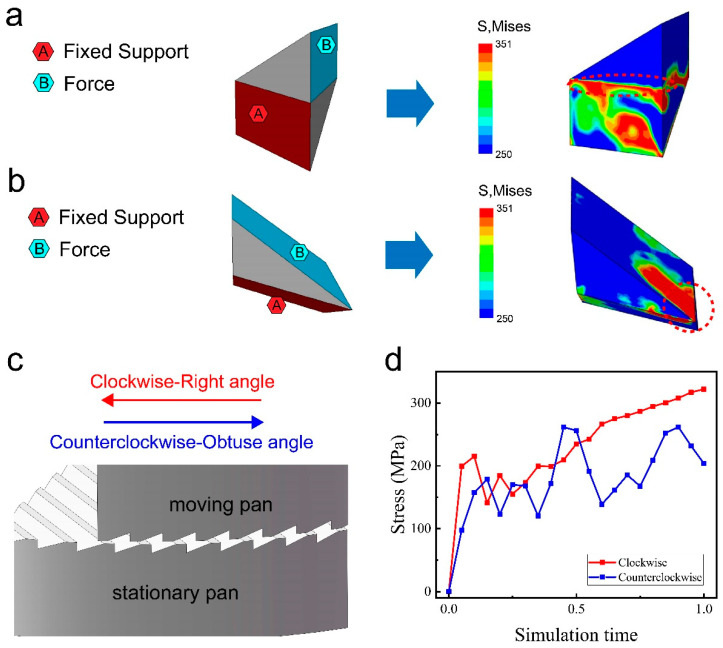
Effect of rotation direction on Mises stress. Stress clouds for (**a**) right-angle cut and (**b**) obtuse-angle cut. (**c**) Diagram of the direction of rotation of the mill-pan. (**d**) Variation in the average Mises stress with time for different pan-milling directions.

The unique structure of the milling teeth results in different pan-milling effects in different rotational directions [30] (Figure 6a,b). A clockwise rotation of the moving pan indicates that the two right-angled edges of the upper and lower mill-pan will cut the model, whereas the counterclockwise rotation indicates the two obtuse edges of the upper and lower mill-pan will cut the model (Figure 6c). The different cutting methods lead to different primary support and stress application surfaces on the model. The maximum stress in the two different cutting methods converges at the junction region of the different support and force surfaces (red circle area in Figure 6a,b). The finite element analysis further corroborates that the right-angle cut method has more finite element meshes reaching the yield stress threshold compared to the obtuse-angle cut, indicating its better stress transfer efficiency. This result is further substantiated by an analysis of the Mises stress across ten randomly selected cells (Figure 6d), which demonstrates that the stress field exerted by a right-angle cut is larger than that of an obtuse-angle cut.

**Figure 7:**
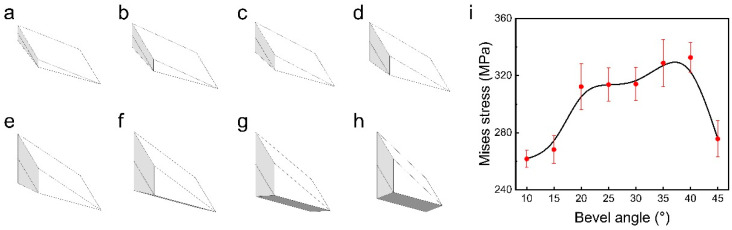
The model of the diamond-shaped block obtained by changing the bevel angle of the mill-pan. (**a**–**h**) correspond to the bevel angle of 10°, 15°, 20°, 25°, 30°, 35°, 40°, and 45°, respectively. (**i**) The relationship between the bevel angle of the mill-pan and the average value of Mises stress on the material.

To investigate the optimal design of the mill-pan structure, we set the bevel angles of the mill-pan to 10°, 15°, 20°, 25°, 30°, 40°, and 45°. These varied angles result in eight diamond-shaped blocks with different shapes and sizes (depicted in Figure 7a–h), which serve to represent the material occupying the unit cell. Ten nodes of each model are randomly selected for the calculation of the average value of the Mises stress under the maximum load, with the results shown in Figure 7i. The comparison of the results for the eight structures shows that the von Mises stress gradually increases with the increase in the bevel angle, which has a peak value between 35° and 40°. Beyond this range, there is a significant decrease in stress. Therefore, in order to maximize the Mises stress, the bevel angle should be set between 35° and 40°.

Previous works [12,31] reported that S3M equipment can achieve mechanochemical desulfurization of vulcanized rubber at room temperature. The experimental results showed a notable decrease in the crosslink density and gel content of vulcanized rubber with the increase in the milling number, alongside a significant enhancement in the mechanical properties of the revulcanized rubber.

S3M equipment can exert enough energy to break the sulfur bonds. However, a critical inquiry pertains to whether the vulcanized rubber within the unit cell could receive sufficient stress from the mill-pan to maximize the breaking of sulfur bonds. As the vulcanized rubber particles are introduced into the unit cell, the upper part of the particles experiences relative displacement against the stationary pan driven by the rotation of the moving pan. In this process, the groove surfaces of both the moving and stationary pans act as the force application surface. The groove of the unit cells needs to have an adequate depth to accommodate the vulcanized rubber particles, ensuring their adherence to the groove surface of both mill-pans. Additionally, the condition of the prong edges is also crucial during the tearing process. Sharp edges may cut off the material of the moving pan during its relative movement or while passing through the intersection of the prongs of both pans. To break the cross-linked bonds of the vulcanized rubber, the rubber particles must undergo a large deformation to reach the necessary distance for the breakage of sulfur bonds. Thus, it is desirable for the edges of the prongs to have small, rounded corners (too large a rounded corner will not effectively apply force to the material). Furthermore, proper clearance between the abrasive discs is required to provide the space needed for the tensile deformation of the vulcanized rubber.

**Figure 8:**
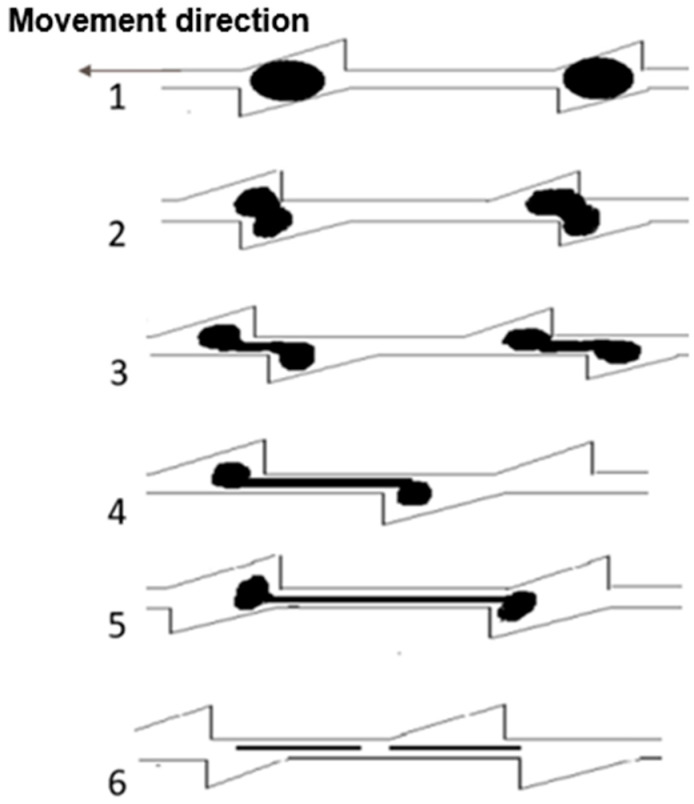
Schematic diagram of the breakage of rubber powder particles in mill-pan.

As deduced in the previous analysis, the inclination angle should be set between 35 and 40 degrees to maximize the equivalent von Mises stresses within the crushed material under the same conditions. Therefore, the slot surface inclination angle was adjusted to 35 degrees for the structure optimization. The quintessential slot configuration should permit exactly half of the gum powder particles to be exposed—that is, the slot’s depth should equal half the size of the gum powder particles (Figure 8). Due to the close fit of the upper and lower mill-pans, the prongs of the disc drive the rubber powder particles to elastic deformation until they are torn off as the moving pan rotates. Therefore, to achieve an optimal processing efficiency, the width and depth of the groove should be comparable to the monomer size of the processed material.

Considering the irregular shape of the material, the width of the slot is set to be 5% larger than the monomer size of the material according to the previous analysis. Therefore, it is possible to calculate the optimum structural dimensions of the trough (right-angled triangular structure). Table 3 shows the corresponding chute dimensions for different material particle sizes.

After the material enters from the center, it repeatedly passes through subsequent unit cells, becoming gradually smaller or deformed after processing in the initial cells. It is evident that the devulcanization efficiency decreases in these later unit cells. Moreover, it is possible that multiple vulcanized rubber particles are accumulated within a single unit cell. Thus, even smaller particles can be effectively milled. Consequently, the overall size of the slot can be appropriately reduced in the design of the slot structure.

**Figure 9:**
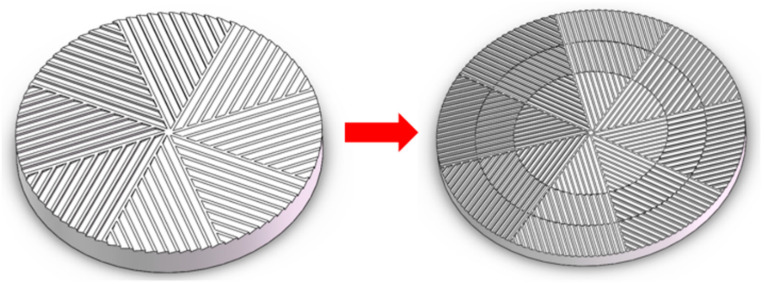
Single-stage inlaid pan-mill and multi-stage inlaid pan-mill.

Vulcanized rubber particles of different sizes should be processed in mill-pans of the corresponding size to obtain the optimum devulcanization results. Once a particular size of material undergoes milling to achieve devulcanization, a mill-pan with the identical structure will diminish desulfurization effects in subsequent processing. Therefore, it is suggested to design a multi-stage mill-pan structure in which the groove size gradually matches the average size reduction in the vulcanized rubber particles after milling. The multi-stage structure design ensures high processing efficiency as the material becomes smaller after initial processing. As shown in Figure 9, unlike the single-stage mill-pan, which has the same spacing and the same depth of groove, the multi-stage mill-pan is divided into three levels from the inside to the outside. Each level maintains consistent spacing and depth within itself, but these dimensions gradually decrease across successive levels, accommodating the progressive size reduction in the processed material.

## 4. Conclusions

In this paper, we study the structure of S3M equipment and its working process and mechanism of crushing materials by the finite element method, proposing a model simplification strategy to optimize calculation time without compromising accuracy. Finite element analysis demonstrates that the Mises stress exerted on the material during pan-milling exceeds the yield stress of most polymeric materials, elucidating the efficacy of the S3M equipment in pulverizing polymeric materials at room temperature. The experimental results further demonstrate that S3M equipment can be used for the preparation of ultrafine micro/nano powders from large polymer and metal particles. The results of the stress analysis show that the shear stress is significantly greater than the positive stress during pan-milling, which is the primary contributor to the fragmentation of the material. It is also proved that the milling effect is better with right-angle cutting. Maximum Mises stress is achieved when the bevel angle of the mill-pan is set between 35° and 40°. Furthermore, we demonstrate that S3M equipment is also valid for rubber devulcanization. Vulcanized rubber particles undergo large tensile deformation with the synergistic effect of the upper and lower mill-pans during the grinding process, which promotes the breakage of sulfur bonds through intermolecular separation. According to the evaluation, the size of the mill-pan groove needs to adequately match the size of the vulcanized rubber particles to achieve optimum processing efficiency. Based on this principle, the multi-stage structure of the milling disc is designed to maintain high processing efficiency for the secondary milling of the material, which theoretically enhances the devulcanization effect.

## Figures and Tables

**Table 1:** Structural parameters of mill-pan.

Parameters	Numerical Values
Radius, R (mm)	50
Division number, n	8
Slot number, m	10
Slot top width, δ (mm)	1
Bevel angle, α (°)	25

**Table 2:** Mechanical parameters of the material [25].

Materials	Young’s Modulus (MPa)	Yield Strength (MPa)
Polystyrene	3600 (Data from [26])	30.9
Iron	211,000 (Data from [27])	220
316L stainless steel	200,000 (Data from [28])	220~170

**Table 3:** Slot structure corresponding to material size.

Particle Size (mesh)	Particle Size (mm)	Bevel Angle (°)	Slot Width (mm)	Slot Depth (mm)	Slot Ramp Length (mm)	Rib Width (mm)
2	8	35	10.97	7.68	13.40	70
5	3.9	35	5.53	3.75	6.53	24.46
8	2.4	35	3.29	2.31	4.02	14.88
10	1.7	35	2.33	1.63	2.85	9.26
20	0.83	35	1.14	0.80	1.39	7.26
30	0.55	35	0.75	0.53	0.92	4.81
40	0.38	35	0.52	0.36	0.64	3.33
50	0.27	35	0.37	0.26	0.45	2.36
80	0.18	35	0.25	0.17	0.30	1.58
100	0.15	35	0.21	0.14	0.25	1.31
150	0.106	35	0.15	0.10	0.18	0.93
200	0.075	35	0.10	0.07	0.13	0.66
300	0.048	35	0.07	0.05	0.08	0.42
500	0.025	35	0.03	0.02	0.04	0.22
600	0.023	35	0.03	0.02	0.04	0.2
800	0.018	35	0.02	0.02	0.03	0.16
1000	0.013	35	0.02	0.01	0.02	0.11

## Data Availability

The original contributions presented in the study are included in the article/Supplementary Materials, further inquiries can be directed to the corresponding authors.

## Supplementary Materials

The following supporting information can be downloaded at: https://www.mdpi.com/article/10.3390/ma17174210/s1.
