# Supplementary material for: Finite Element Analysis of the Structure and Working Principle of Solid-State Shear Milling (S3M) Equipment
Source: Materials (Basel). 2024 Aug 26;17(17):4210. doi: 10.3390/ma17174210 (PMC11396375; doi:10.3390/ma17174210)
Supplement: Supplementary file 1 [file materials-17-04210-s001.zip › materials-3093543-supplementary.pdf]

Modeling process

The center of the circle of the mill-pan is the location of the feeding port of the solid-state shear milling equipment. The unit cells around the feeding port are easy to fill and accumulate particles because of the low linear velocity and fast feeding rate. Therefore, we consider the particles inside a unit cell near the inlet as a whole and analyze the forces. The structural parameters of the mill-pan are shown in Table 1. The 3D model of the mill-pan with different structures can be obtained by adjusting these important parameters. The process of unit cell creation is shown in Figure S1. The material model between the two mill-pans is obtained by Boolean operation of the 3D graph. Based on the 8-fold symmetry of the material model and reducing the computation time, only one-eighth of the material was modeled. The model of the diamond-shaped block near the center of the mill-pan is intercepted by segmentation and used as the model for this finite element analysis. After reasonable model simplification, the difficulty of finite element calculation is greatly reduced.

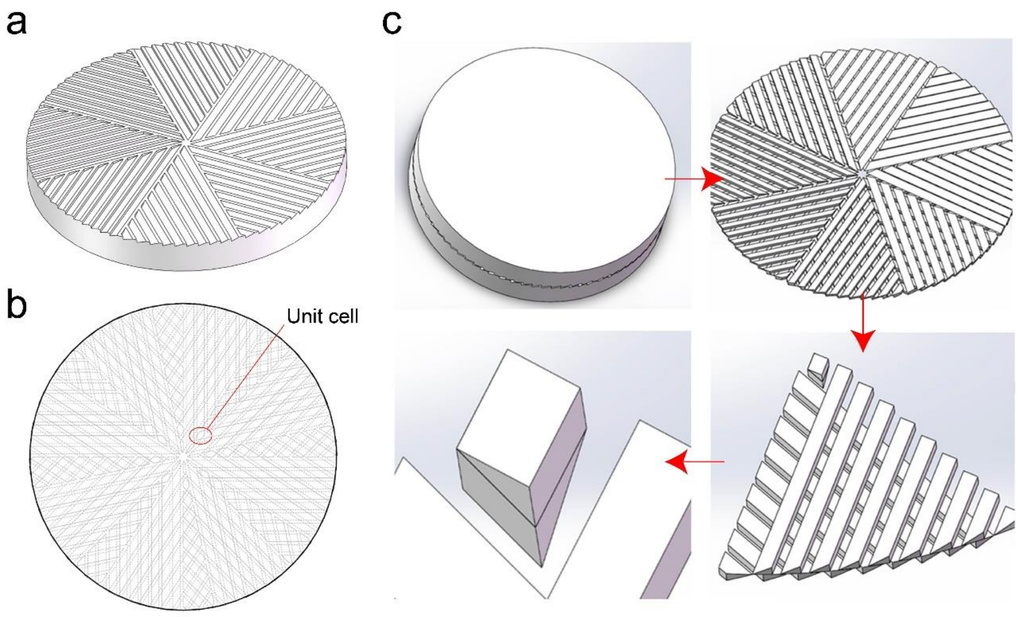

Figure S1. Construction of the 3D model. (a) 3D model of the mill-pan. (b) The shape of the unit cell between the upper and lower mill-pans as seen from the top view. (c) The process of establishing the diamond-shaped block model.

Details of finite element analysis

The three-dimensional finite element model and mesh are shown in Figure S2a-b, which features a deformable diamond-shaped block and simplifies the mill-pan into two sets of rigid surfaces. Eight-node brick elements with three translation degrees of freedom at each node (C3D8R) were used in the mesh discretization of the deformable body. Surface-to-surface contact is used to simulate the interactions between the inner surface of the mill-pan and the outer surface of the material, with defined contact surface pairs facilitating this interaction. “Hard contact” in the normal direction can be specified for the interface, which allows for the separation of the interface in tension and no penetration of that in compression. The tangential interaction contact can be simulated by the Coulomb friction model, necessitating refined mesh near the contact zones for numerical precision. The complicated contact relationships between the inner surface of the mill-pans and the outer surface of the material require finer meshing. Therefore, despite the increased computation time, we divide the whole deformable body into a more detailed mesh (Figure S2b) to ensure the accuracy of the numerical computation. The mesh was well-tested for convergence, i.e., further refinement of the mesh size would not improve the accuracy of the simulated results. The material is set to a carbon steel material AISI 1020, which is commonly used in finite element simulations. Its modulus of elasticity is 200000 MPa, Poisson's ratio is 0.29, and yield stress is 351.571MPa, respectively, which exceeds most polymer materials. Different researchers have used different stress–strain models for this type of steel material [1, 2]. The ideal elastoplastic model (Figure S2c) was used to simplify the stress–strain curves and the observation of yield limit attainment. Unconstrained in its translational and rotational degrees of freedom, the material navigates within the gap between the two mill-pans with the rotation of the moving pan. To contrast, the stationary pan is set to be completely fixed with constrained translational and rotational degrees of freedom. In the coordinate system shown in Figure S2a, the moving pan can only rotate around the y coordinate axis, limiting its translational and two rotational degrees of freedom. The results of the finite element analysis show that the trends of the Mises stress and

equivalent variation (Figure S2f) of the nodes on the material fit well with the ideal elastoplastic model (Figure S2c).

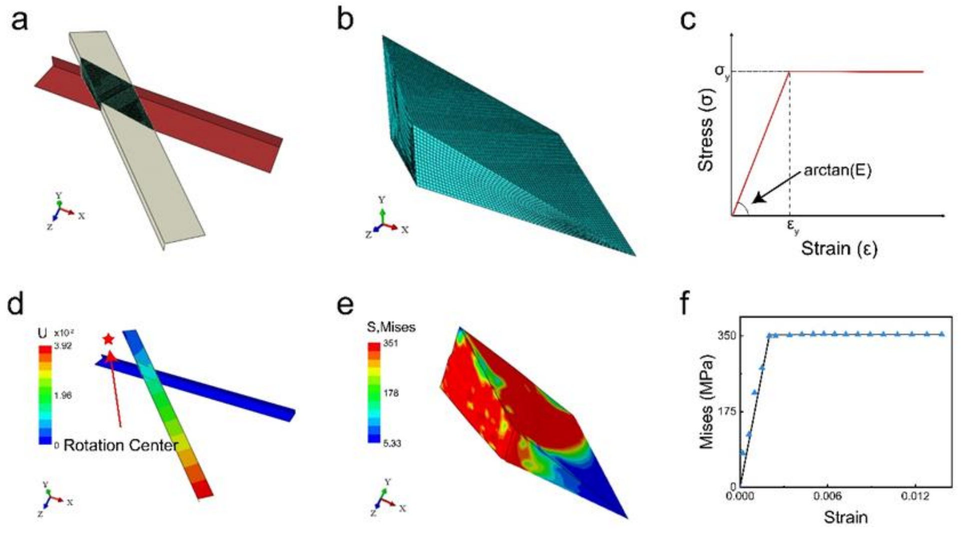

Figure S2. Finite element model and calculation results. (a) Overall model and mesh for finite element analysis. (b) Refined mesh for diamond-shaped block. (c) Stress–strain curve of the ideal elastoplastic model. Displacement diagram (d) and von Mises stress field (e) of AISI 1020 under maximum load in finite element simulation. (f) The trend of Mises stresses and strains at the nodes in the finite element model.

Explicit dynamics method

Implicit methods require very small time increments to accurately solve the physical phenomena involved when simulating complex engineering problems. The process of implicit method computation requires continuous iterations to solve the system of equations, which will lead to the problem of convergence. Conversely, the explicit method uses dynamic equations in differential format without directly solving the stiffness matrix or performing equilibrium iterations, which is less time-consuming and computationally inexpensive [3, 4]. Therefore, the explicit method is employed for this simulation. At the beginning of the incremental step, the acceleration of each node is solved by a time-centered difference based on the following dynamic equilibrium equation:

$$\ddot{\mu}|_t = M^{-1}(P - I) \quad (S1.),$$

where M, $\ddot{\mu}$, P, and I are the nodal mass, acceleration, external force, and internal force, respectively. A time-centered difference is applied to calculate the nodal velocities:

$$\dot{\mu}\big|_{t+\frac{\Delta t}{2}} = \dot{\mu}\big|_{t-\frac{\Delta t}{2}} + \ddot{\mu}|_t \frac{\Delta t|_{t+\Delta t} + \Delta t|_t}{2} \quad (S2.).$$

After obtaining the node velocity $\dot{\mu}$, the node displacement μ is expressed as follows:

$$\mu|_{t+\Delta t} = \mu|_t + \dot{\mu}\big|_{t+\frac{\Delta t}{2}} \Delta t|_{t+\Delta t} \quad (S3.).$$

The acceleration, velocity, and displacement of the node at moment t could be obtained according to these three equations. The strain rate $\dot{\varepsilon}$ of the cell is used to measure its strain increment tensor dε. The stress σ in the cell is solved by the material's instanton equation $\sigma|_{t+\Delta t} = f(\varepsilon, \dot{\varepsilon}, T)$, and the internal force $I_{t+\Delta t}$ in the cell node is integrated to complete the calculation of all data at moment t. After that, the time goes forward Δt, and the data of the solution node and cell at the moment t+Δt are solved.

von Mises yield criterion

Mises stress can be calculated from the three principal stresses in each cell. In the principal stress coordinate system of the cell, the bias stress tensor is zero, i.e., only the principal stresses σ_1, σ_2, and σ_3 exist in the direction along with this coordinate axis. Mises yield condition is only related to the second bias stress tensor invariant, which can be expressed as follows:

$$J_2 = \frac{1}{2}\zeta_{ij}\zeta_{ij} = I_2 + 3\tau_m^2 \quad (S4.),$$

where ζ_{ij} is the component of the partial stress tensor, I_2 is the second invariant, and τ_m is the mean positive stress,

$$I_2 = \sigma_1\sigma_2 + \sigma_2\sigma_3 + \sigma_3\sigma_1 \quad (S5.),$$

$$\tau_m = \frac{1}{3}(\sigma_1 + \sigma_2 + \sigma_3) \quad (S6.).$$

After constant transformation, the second partial stress tensor invariant can be calculated from the second invariant and the average positive stress.

Mathematically, the von Mises yielding criterion is expressed as follows:

$$J_2 = k^2 \qquad (S7.),$$

where k is the yield stress of the material in pure shear. At the onset of yield, the magnitude of the shear yield stress in pure shear is $\sqrt{3}$ times greater than the tensile yield stress in the simple tension case:

$$k = \frac{\sigma_y}{\sqrt{3}} \qquad (S8.),$$

where σ_y is the tensile yield strength of the material measured by uniaxial tensile tests. The von Mises yield criterion can be expressed according to the aforementioned equations:

$$\sigma_y = \sqrt{3J_2} \qquad (S9.).$$

The finite element calculation enables the determination of the stress state for each unit on the material and helps the calculation of Mises stress values. The comparison with the yield stress value of the material could be used to determine whether the unit has yielded or not.

References

[1] Z. Tao, Z.-B. Wang, Q. Yu, Finite element modelling of concrete-filled steel stub columns under axial compression, Journal of Constructional Steel Research, 89 (2013) 121-131.

[2] M. Dundu, Evolution of stress–strain models of stainless steel in structural engineering applications, Construction and Building Materials, 165 (2018) 413-423.

[3] T. Elguedj, A. Gravouil, H. Maigre, An explicit dynamics extended finite element method. Part 1: Mass lumping for arbitrary enrichment functions, Computer Methods in Applied Mechanics and Engineering, 198 (2009) 2297-2317.

[4] A. Gravouil, T. Elguedj, H. Maigre, An explicit dynamics extended finite element method. Part 2: Element-by-element stable-explicit/explicit dynamic scheme, Computer Methods in Applied Mechanics and Engineering, 198 (2009) 2318-2328.
